# Atypical forms of pityriasis versicolor: Case series and literature review

**DOI:** 10.1016/j.jdcr.2025.06.047

**Published:** 2025-07-24

**Authors:** Francesca Gaudiello, Nello Tommasino, Silvia D’Ascenzo, Massimiliano Scalvenzi, Luciano Foggia

**Affiliations:** Section of Dermatology, Department of Clinical Medicine and Surgery, University of Naples Federico II, Naples, Italy

**Keywords:** atypical, childhood, face, forms, Malassezia, mycosis, pityriasis versicolor, tinea

## Introduction

Pityriasis versicolor (PV) occurs more frequently in areas with higher temperatures and higher relative humidity.^1^ Its prevalence is statistically different between tropical areas and Scandinavia (50% vs 1%).^2^ Prevalence increase has been registered in Italy, probably due to ecosystem tropicalization.

Currently, there is an increased incidence of recurrent forms of PV. These may include atypical variants in terms of age of onset, extent and appearance. To identify PV subtypes, cell cultures and PCR are difficult techniques to employ, so the diagnosis still remains clinical. The therapeutic management of atypical forms is challenging. One or more significant cases are reported for each atypical form of PV. The aim of this paper is to describe the clinical features of these atypical forms to increase knowledge and improve management of these difficult cases.

## Methods

Several cases of atypical forms of PV that came to the attention of the authors have been described. A literature search was subsequently conducted on the Pubmed, EBSCO, Google Scholar, Embase, and Cochrane Skin, by querying the terms: 'tinea,' 'versicolor,' 'pityriasis,' 'atypical,' 'forms,' 'variants' (until May 2024). Only English language manuscripts were considered, which included reviews, meta-analyses, case reports, case series, and real-life studies.

## Atypical clinical forms

### Infant forms

PV mostly affects adolescents and young adults, while its occurrence in infants under 2 years of age is uncommon.^2^ This is due to immaturity of sebaceous glands and lower production of sebum, which serves as a source of Malassezia.^2^ In infants, clinical manifestations are different from those seen in adolescents and adults. In these patients PV is more inflammatory, lesions are more extensive, localized not only on the trunk but also on the face, spread rapidly, and are more difficult to treat.^3^

**Fig 1:**
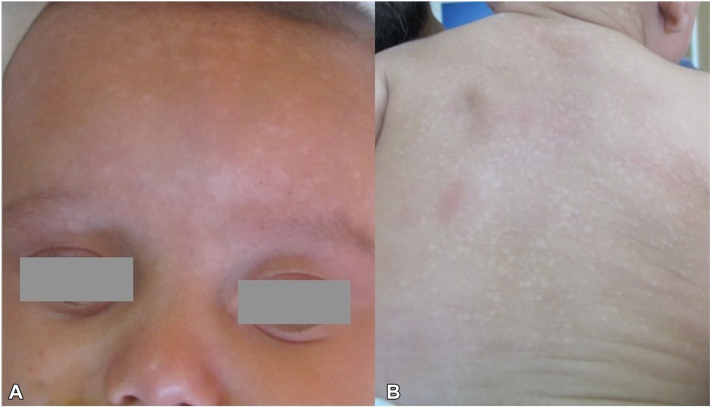
PV of the infant. **A,** Hypopigmented and hyperpigmented macules of the face distributed all over the forehead; **(B)**: hypopigmented and hyperpigmented macules of the upper back. *PV*, Pityriasis versicolor.

A case of a 4-month-old male baby was examined for the occurrence of depigmented areas on his trunk, scalp and face that were noticed at the age of 2 months. The patient had been carried at term and was born without complications. On physical examination, numerous hyper- and hypopigmented macules were detected on the scalp, face, shoulders and upper back (Fig 1). Microscopic examination of scrapings from the lesions on the shoulder revealed spherical yeast forms and hyphae that were broken up into short filaments, that confirmed the diagnosis of PV. He was then treated with topical clotrimazole 1% solution twice daily for 4 weeks, with marked clinical improvement.

The rare cases in the literature of PV in children involve male children between 2 and 8 months of age from tropical countries (mainly India). Premature infants are more often found to have yeast on their skin, regardless of whether they develop lesions. One of the hypothesized causes may be the more frequent management of these children by health care personnel than babies born at term and receive lipid supplementation via catheters.^2^

### Facial localization

**Fig 2:**
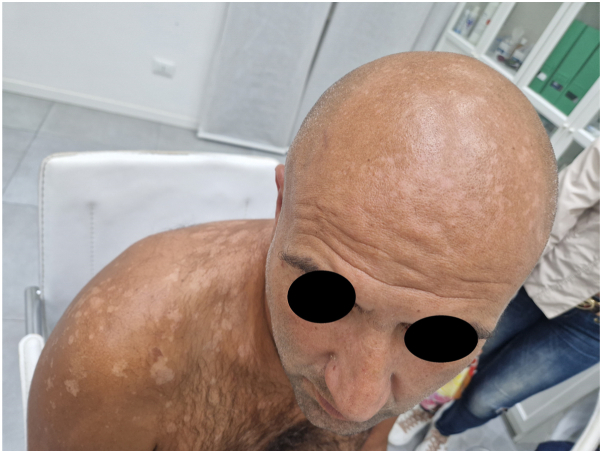
Facial involvement of PV. Hypopigmented and hyperpigmented macules of the scalp, shoulder and upper back, larger in size than the infant form. *PV*, Pityriasis versicolor.

Although localization to the face is more common in childhood, in adults is quite common in tropical and subtropical regions, where it occurs in more than half of patients.^4^ Climate change with rising temperatures is supposed to be cause of the recent increasing cases of pityriasis versicolor with facial localization. Increased sweating of the face may be a predisposing factor for facial development of Malassezia. In addition, this yeast on the face of dark-skinned individuals can cause petaloid forms of PV/seborrheic dermatitis.^5^ Also in Italy, we described the case of a 54-year-old patient who had involvement of the back and scalp in addition to the face (Fig 2). The clinical suspicion was confirmed by microscopic examination, and therapy with topical ketoconazole 2% was undertaken.

### Extended forms

**Fig 3:**
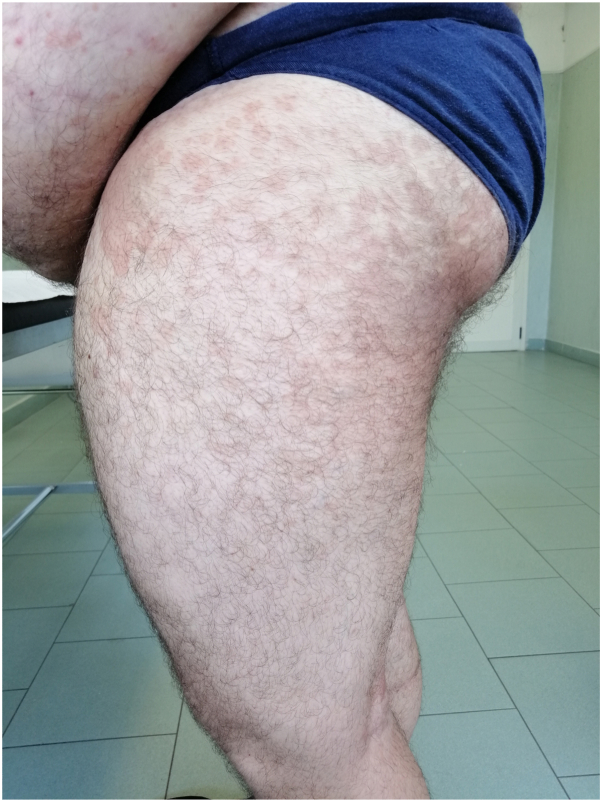
Extended form of PV in a 54-y old man. Hypopigmented and hyperpigmented macules extended to the distal end of the thigh. *PV*, Pityriasis versicolor.

**Fig 4:**
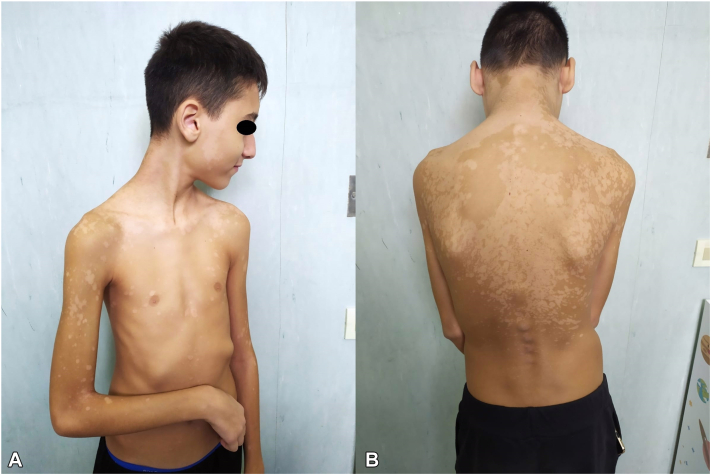
Extended form of PV in a 13-years old boy. **A,** Hypopigmented patches involving atypical sites such as the face and upper limb up to the forearm, as well as the trunk. **B,** The same manifestations expressed massively at the back as well. *PV*, Pityriasis versicolor.

Recently, extensive forms of pityriasis versicolor are increasing. A 54-year-old man presented to our dermatology clinic for the appearance for about 2 months of hyperpigmented patches localized at the trunk, abdomen, proximal region of the thighs, and popliteal cord, with itching and chronic relapsing disease trend for about 10 years (Fig 3). A microscopic examination was performed, which showed the “spaghetti-and-meatball” appearance, typical of Malassezia. As the lesions were atypically localized on the lower limbs, a cultural examination was subsequently performed, which demonstrated the presence of multiple subspecies of Malassezia. The patient was a farmer and was exposed to the sun for many hours a day, resulting in hyperhidrosis. This condition is related with an increased susceptibility to PV, a growing risk of larger lesion size and an increased rate of recurrence.^6^ In fact, the yeast feeds on the amino acids present on the skin surface such as glycine and higher concentrations of glycine have been demonstrated in the sweat of subjects with hyperhidrosis.^7^ A similar case was observed in a 13-year-old boy with extension of PV to most of the body surface, including the face, neck, back and lower limbs (Fig 4). The patient's history was characterized by high sweat production, secondary to pubertal change and his sports activity, as he played tennis for 4 hours a day. These cases indicate that PV should not be ruled out if the localization of occurrences is atypical.

### Hyperkeratotic forms

**Fig 5:**
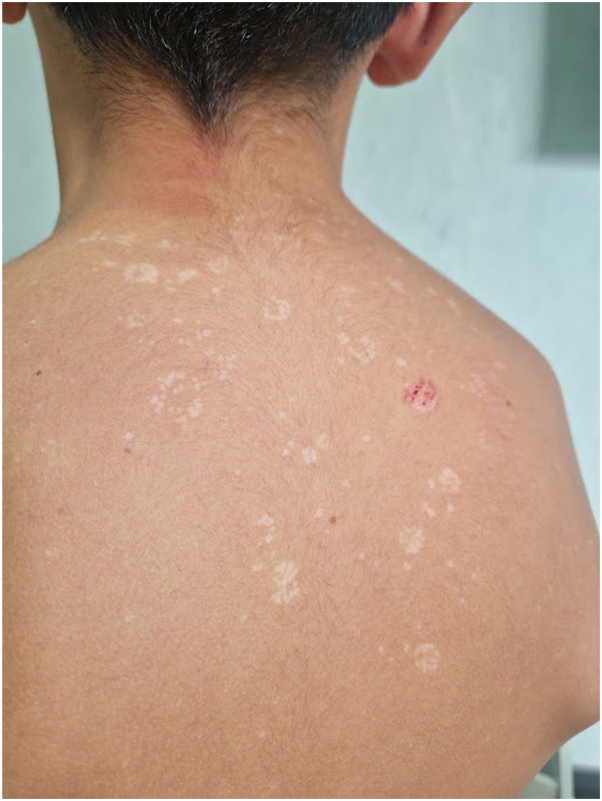
Hyperkeratotic form of PV in a 13-years old boy. The macules are characterized by hyperkeratosis lace at the periphery that gave a garland-like appearance. *PV*, Pityriasis versicolor.

**Fig 6:**
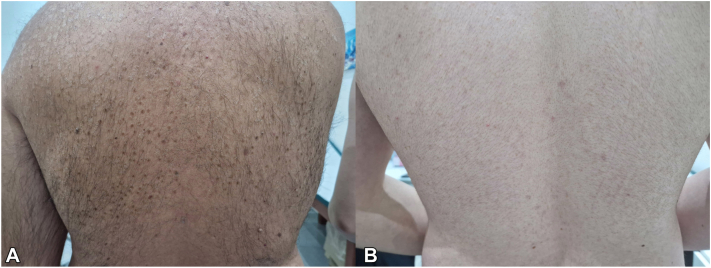
Hyperkeratotic form of PV in an adult and his son. **A,** manifestations of the back of the father, again with garland-like periphery surrounding a fine central desquamation. **B,** Lesions of the son in the same localization with same appearance. *PV*, Pityriasis versicolor.

**Fig 7:**
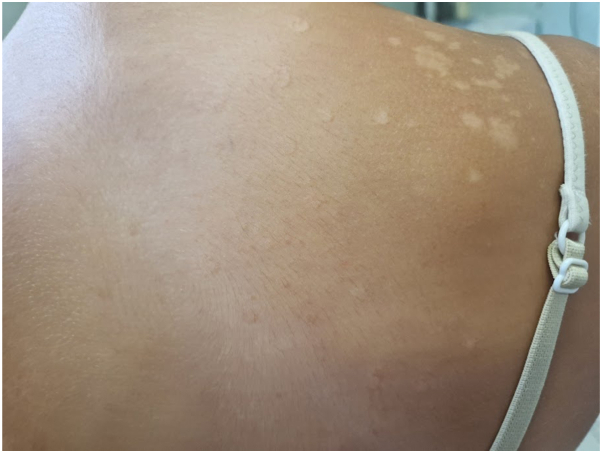
Hyperkeratotic form of PV in a 9-years old girl. Back exhibits macules with peripheral hyperkeratosis, while hypopigmented macules are observed on the right shoulder. *PV*, Pityriasis versicolor.

Four cases of back PV were then examined, in a 13-year-old boy (Fig 5), in the same family where father and son shared the same type of PV (Fig 6), and in a 9-year-old girl where 2 different forms of PV were observed (Fig 7). In our patients, the lesions had a fine hyperkeratosis lace at the periphery that gave a garland-like appearance. The keratolytic activity of yeast may be due to mechanical or chemical degradation of keratin; the keratin content of cells invaded by Malassezia may be largely replaced by an amorphous, lipid-dense material as a result of the action of keratinase.^8^ In addition, lipid-like droplets may be present among the keratinocytes. One possible explanation could be sought among the various subtypes of Malassezia, which might have different keratinase activity and give rise to this form of PV. However, this does not affect the prognosis of the condition, as these forms resolved with 20 days of topical clotrimazole 1% therapy.

### Annular hyperpigmented forms

**Fig 8:**
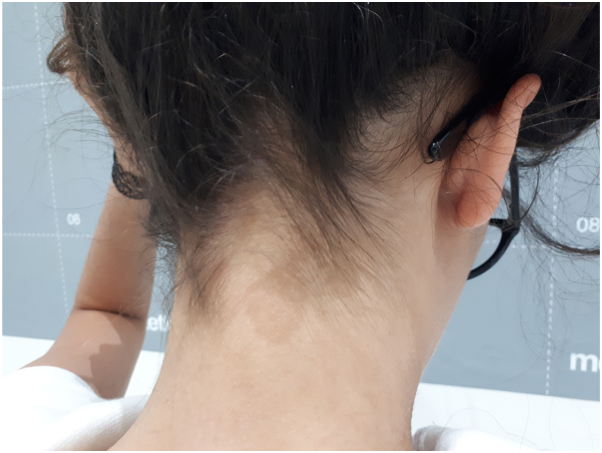
Annular hyperpigmented form in a 41-years old woman. The nape of the neck shows finely scaling hyperpigmented annular macules.

A 41-year-old woman with no comorbidities presented to our dermatology clinic for the appearance for about 1 month of small annular hyperpigmented macules localized on the neck (Fig 8). The macules were finely scaling and did not cause itching but great esthetic discomfort for the patient. After therapeutic failure with topical corticosteroids, microscopic examination by KOH staining was performed and confirmed pityriasis versicolor. The same manifestations affected the neck of a 19-year-old woman and the back of a 27-year-old woman. In both cases, the manifestations arose rapidly and the resolution had been equally rapid. Moreover, no recurrences had occurred between 3 and 5 years. This is probably a characteristic feature of this form of PV, which differ from the typical ones that tend to recur frequently.

The macules and patches that characterize PV can be either hypopigmented (particularly clear in dark-skinned subjects) or hyperpigmented, in which case the coloration varies from pink to dark brown. Hyperpigmented lesions appear to contain more spores and hyphae than normal or hypopigmented skin. The fungus may cause differentiation of Merkel cells from epidermal cells; these may have an increased activity because they contain compound melanosomes and secretory granules.^2^ The appearance of melanocytes in hyperpigmented PV lesions is also altered; they may be hypertrophic and are more likely to be singly distributed.^9^ Depigmentation has been explained by the production of dicarboxylic acids produced by Malassezia species (eg, azelaic acid) that cause competitive inhibition of tyrosinase and perhaps a direct cytotoxic effect on overactive melanocytes.^8^ M. furfur produces pigments and fluorochromes with tryptophan as the only nitrogen source.^10^ They (ie, malassezine, pityriacitrin, pityrialactone, pityriarubin) may explain some clinical phenomena of pityriasis versicolor (depigmentation, fluorescence, absence of sunburn) in PV alba.^11^ Irregular pigmentation within a lesion is the most common dermoscopic feature observed in both subjects with hypopigmented and hyperpigmented macules.^12^ PV lesions generally do not have a clearly demarcated border separating them from normal skin, unlike the clearly demarcated lesions of vitiligo.^12^ PV lesions appear to distribute radially from the hair follicles and, as they progress, merge with other lesions to form different shapes.^12^ At histology, hyperpigmented lesions have a greater number of organisms and a more pronounced inflammatory infiltrate than hypopigmented lesions.^8^ In addition, a more widespread type of desquamation has been observed more frequently in hyperpigmented lesions than in hypopigmented lesions.

## Discussion

Malassezia are lipid-dependent yeasts that colonize the skin of humans and other warm-blooded animals, immediately after birth. They usually occur as skin commensals, but are also associated with various skin disorders (such as head & neck dermatitis, seborrheic dermatitis, Pityriasis versicolor and Malassezia folliculitis).^10^ What distinguishes PV is the transformation from the saprophytic to the parasitic form, characterized by the appearance of pseudomycelium.^13^ In other Malassezia dermatoses, the underlying mechanisms are different, such as colonization of the bulb in folliculitis or increased production of inflammatory cytokines in response to parasite growth seen in seborrheic dermatitis.^10^

In such cases metabolic product as Malassezia indole derivatives have immunosuppressive effect with clinical manifestation without inflammation (ie PV); in other cases (ie Seborrheic dermatitis) Malassezia metabolic products and surface components act as irritant non-immunogenic stimulation of the immune system that leads to complement activation and local increase in NK1+ and CD16+ cells.^14^ Malnutrition, poor general health, steroid therapy, and family history of PV are important predisposing factors for PV.

The genus currently comprises 18 species and it seems that multiple Malassezia species and/or genotypes may cause unique pathologies configuring atypical forms with reduced antifungal susceptibility.^3^ The most frequently observed species are M. furfur, M. globosa and M. sympodialis.^15^ In vitro identification of the different species of Malassezia can be difficult. The techniques employed are based on the study of colony morphology, macro- and microscopic, catalase and beta-glucosidase reaction, and the study of the pattern of requirement or assimilation of different lipids, namely: Tweens 20, 40, 60, and 80 and of cremophor EL (castor oil).^16^

Clinical routine implies direct exams by KOH processing and microscopic observation, but it could be necessary typifying the species by cellular cultures and/or PCR in order to predict skin interaction pattern and eventually antifungal susceptibility. For example, Mirhendi et al used a PCR-based technique with restriction enzyme digestion for discrimination of 11 Malassezia species.^17^ Isolating, maintaining, and identifying these yeasts is challenging, but recognizing physiological aspects such as the presence of catalase or pigment production can help in the targeted treatment of atypical forms such as those described.^18^

The increasingly frequent extension and recurrences that we are seeing of PV, could certainly be caused by a number of factors: genetic, iatrogenic, high temperatures, the degree of humidity and occlusive effect of clothing and especially sweating as mentioned above. But the new atypical clinical forms, also found in recent years in Italy, may have their explanation in the spread of new subtypes of Malassezia from tropical regions. Moreover the isolation of more than one species in a single lesion is not uncommon in PV and is related to the presence of a predisposing factor.

In clinical practice Malassezia infection and related skin disorders are widely increasing and that could imply clinicians' concern about possible local and/or systemic complications. We need further studies to better diagnose, treat and manage PV and Malassezia related clinical presentations. Lipid-containing infusions used in neonatal intensive care have been described among the risk factors for systemic Malassezia infections.^19^ Other risk factors include immunosuppression, such as HIV infection and prolonged corticosteroid therapies, and hematologic malignancies.^19^ The resulting pathology and frail condition of these patients increase their mortality rate. Mention should also be made of atrophying PV, a rare variant characterized by rounded lesions with a depressed appearance. Although the etiology of this form is unknown, a role of topical corticosteroids incorrectly prescribed following diagnosis of eczema is speculated.^20^ The diagnosis of systemic Malassezia infection is not simple. In fact, although blood culture is the gold standard, specific cautions must be used, such as lipid supplements in the cultures and a prolonged incubation period at 2 weeks.^19^ The first line of treatment for PV is topical antifungals. These are very safe and less expensive drugs than systemic drugs. They are represented by azoles, naftifine, ciclopiroxolamine and terbinafine, available in cream, shampoo, foam, gel or lotion formats. The most reported adverse effects are irritation and contact allergy.^8^ Systemic antifungals are indicated in cases of massive extension of PV or after failure of topical therapy. Itraconazole and oral fluconazole are the systemic drugs used in these cases, but also ketoconazole is effective. However, the risk of hepatotoxicity limits its use. In contrast, oral terbinafine and griseofulvin are not effective against PV.^8^

In conclusion, atypical forms of PV are increasing, probably due to the tropicalization of the climate. Diagnosis of these forms is challenging because of the complex differential diagnosis that often requires exams that are not easy to perform such as culture and PCR. Further studies are needed to improve the diagnostic capabilities of these forms and the use of the mentioned techniques.

## Conflicts of interest

None disclosed.
